# Biomarkers of Pre‐COPD Lung Function Trajectories in Middle Age: A Prospective Study From 1st to 6th Decade

**DOI:** 10.1002/resp.70279

**Published:** 2026-07-13

**Authors:** Dinh S. Bui, Don Vicendese, Jonathan Pham, Nur Sabrina Idrose, Adrian Lowe, Caroline Lodge, Raisa Cassim, Chamara Senaratna, Gayan Bowatte, Garun Hamilton, Alan James, Paul Thomas, Richard Wood‐Baker, Peter Frith, Gary Anderson, Philip M. Hansbro, Graeme Zosky, George Washko, Michael J. Abramson, Rosa Faner, Alvar Agusti, Jennifer Perret, E. Haydn Walters, Shyamali C. Dharmage

**Affiliations:** ^1^ Allergy and Lung Health Unit, School of Population and Global Health The University of Melbourne Melbourne Australia; ^2^ Department of Lung, Sleep, Allergy & Immunology Monash Health Clayton Victoria Australia; ^3^ Department of Pulmonary Physiology and Sleep Medicine Sir Charles Gairdner Hospital Western Australia; ^4^ Inflammation and Infection Research, Faculty of Medicine University of New South Wales New South Wales Australia; ^5^ School of Medicine University of Tasmania Hobart Tasmania Australia; ^6^ College of Medicine and Public Health Flinders University Adelaide Australia; ^7^ Centre for Lung Health University of Melbourne Melbourne Australia; ^8^ Centre for Inflammation, Centenary Institute and University of Technology Sydney Faculty of Science, School of Life Sciences Sydney New South Wales Australia; ^9^ University of Tasmania Hobart Tasmania Australia; ^10^ Harvard Medical School Harvard University Boston Massachusetts USA; ^11^ School of Preventive Medicine Monash University Melbourne Australia; ^12^ Centro de Investigación Biomédica en Red Enfermedades Respiratorias (CIBERES) Barcelona Spain

**Keywords:** biomarker, COPD, lung function trajectory, obstruction, pre‐COPD

## Abstract

**Background and Objective:**

Identifying impaired lifetime lung function trajectories and their underlying biological pathways is key to characterising the preclinical phase of COPD (Pre‐COPD) and its progression. However, this is challenging in practice as repeated spirometry measurements from childhood are rarely available. We investigated potential blood biomarkers associated with pre‐COPD trajectories.

**Methods:**

The Tasmanian Longitudinal Health Study has unique lung function data from 7 to 53 years, from which lifetime FEV_1_/FVC trajectories were modelled (*n* = 2442). Serum levels of CC16, sRAGE, CRP, ECP, SPD, fibrinogen and a range of cytokines were quantified at 53 years of age. These were related to respective FEV_1_/FVC trajectories. Classification And Regression Tree (CART) analysis was used to investigate biomarker combinations in relation to the distribution of pre‐COPD FEV_1_/FVC trajectories.

**Results:**

Among six FEV_1_/FVC trajectories, three trajectories at increased risk of COPD (pre‐COPD trajectories) were ‘Early Low‐Rapid Decline’, ‘Early Normal‐Rapid Decline’ and ‘Early Low‐Normal Decline’. CC16, sRAGE, SPD, IL‐17A, IL‐5 and IL‐10 differed significantly between these three pre‐COPD and the normal trajectory. Notably, within the pre‐COPD trajectories, these biomarkers also differentiated two rapid decline trajectories from the relatively stable one. CART analysis identified different combinations of biomarkers that distinguish different pre‐COPD trajectories.

**Conclusion:**

This is the first study to show that established COPD biomarkers are already active during the pre‐COPD phase and could distinguish pre‐COPD lung function trajectories. Biomarkers, particularly together with clinical history, may have the potential to identify those at risk and optimise management of both Pre‐COPD and COPD.

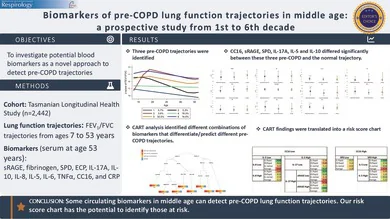

## Graphical abstract

Identifying impaired lifetime lung function trajectories is key to identifying the preclinical phase of COPD (Pre‐COPD) or prognosis of COPD. We have identified that some circulating established COPD biomarkers differ across ‘pre‐COPD’ lung function trajectories, suggesting that some COPD‐specific pathobiological processes, including inflammation, are already active during the pre‐COPD phase. Our findings highlight that some blood biomarkers could be used together with clinical variables in developing predictive algorithms to identify those at risk and optimise management of both Pre‐COPD and COPD in early middle age.

## 1 Introduction

Chronic obstructive pulmonary disease (COPD), a significant public health issue, is now the third leading cause of death, contributing to around 3 million deaths annually worldwide [1]. Accelerated lung function decline was considered the only pathway to COPD. However, the landscape of COPD understanding has changed in the last decade: lung function impairment at other phases/periods across the life course also contributes to COPD development [2, 3]. Indeed, interest in lung function trajectories has increased exponentially in recent years [4]. It is now recognised that multiple different lifetime lung function trajectories can lead to COPD [2, 3].

Among studies that have investigated longitudinal lung function trajectories is the Tasmanian longitudinal Health Study (TAHS), a large population‐based cohort study with repeated lung function measurements that capture both lung function growth and decline phases. TAHS was the first study to demonstrate six population‐based FEV_1_/FVC trajectories that spanned ages 7 to 53 years [5]. Three distinct trajectories were characterised by early low FEV_1_/FVC from childhood and/or rapid FEV_1_/FVC decline in adulthood, and associated with an increased risk of COPD (three ‘pre‐COPD’ trajectories) [5]. Adding to the complexity, these pre‐COPD trajectories included both relatively stable and rapidly progressive decline ones.

Having data on longitudinal lung function trajectories from childhood to early middle age could potentially help predict their risk of future COPD, informing preventive strategies to slow down the development of or prevent progression to COPD in those with a high‐risk trajectory, that is, a looking forward ‘prospective’ concept. Similarly, in patients with spirometrically diagnosable COPD, especially those seen for the first time in clinical practice, knowing their past lung function trajectories could be clinically important for personalised and optimal management, that is, a looking backward ‘retrospective’ concept. This is because COPD stemming from different lung function trajectories may represent different phenotypes with varying prognosis and treatment response. For example, while the disease status in some COPD patients is relatively stable over time, it declines more rapidly in others, and this latter condition is associated with higher rates of acute exacerbations and mortality.

Thus, identifying an individual's lung function trajectory and understanding the potential underlying biological mechanisms may be critical from both public health and clinical perspectives. However, this remains a major challenge as establishing a person's trajectory requires repeated lung function measures beginning from childhood, but such data are rarely available in clinical practice. To address this gap, there is a pressing need to investigate more immediately pragmatic approaches to identify likely lung function trajectories. The use of biomarkers has emerged as a promising approach for providing insights into underlying biological mechanisms and distinguishing disease trajectories [6]. Proteomic signatures have been shown to enhance risk prediction for both common and rare diseases, including the incidence of COPD [7]. However, these studies lack information on the life course trajectories leading to COPD. In turn, cohorts of well‐established COPD, have identified biomarkers of lung function decline and COPD progression such as CC16, sRAGE and SPD, but these studies also lack data on preceding trajectories that led to COPD [8]. CC16 and sRAGE were also found to be associated with COPD phenotypes defined based on lung function decline [9]. We hypothesize that investigating how COPD‐related biomarkers associate with pre‐COPD FEV_1_/FVC trajectories in younger individuals will provide novel insights into early disease development and support the development of targeted preventive and personalised clinical care strategies.

This study aimed to investigate/characterise potential blood biomarkers in pre‐COPD FEV_1_/FVC trajectories with a particular view to identifying whether biomarkers differ between pre‐COPD trajectories and normal controls, as well as between relatively stable pre‐COPD from progressive rapid declining pre‐COPD.

## 2 Methods

### 2.1 Study Population

This analysis used data from the Tasmanian Longitudinal Health Study (TAHS), a large, well‐established, population‐based cohort study [10]. In brief, TAHS began in 1968 when all 8583 children born in 1961 in the State of Tasmania and attending school there were enrolled (99% of the eligible population). At age 7 years (baseline), the children underwent pre‐bronchodilator (BD) spirometry and parents completed a detailed questionnaire. Follow‐up assessments were conducted at ages 13, 18, 45, 50 and 53 years with surveys completed and pre‐BD spirometry measured. In addition, at age 45 years both pre‐ and post‐BD spirometry, single‐breath carbon monoxide diffusing capacity of the lung (D_L_co), and static lung volumes were measured. In the most recent follow‐up (2012–2016), when the participants were 53 years old, all those from the 1968 original cohort who were alive and had up‐to‐date contact details were invited to complete a survey and clinical investigations. In this follow‐up, pre‐ and post‐BD spirometry and D_L_co were performed in 2689 participants.

### 2.2 FEV_1_ /FVC Trajectories

In TAHS, pre‐bronchodilator spirometry was repeatedly measured at six time points from 7 to 53 years of age (7, 14, 20, 45, 50 and 53 years). In those with these repeated measurements of FEV_1_/FVC (*n* = 2422), six distinct trajectories of FEV_1_/FVC were identified using group‐based trajectory modelling (GBTM) [5]. GBTM is a statistical technique that identifies groups of individuals who follow a similar pattern/trajectory of lung function over time. The six identified FEV_1_/FVC trajectories were: Early Low‐Rapid Decline (5.7%), Early Low‐Normal Decline (18.3%), Early Normal‐Rapid Decline (5.3%), Early Low‐Catch up‐Normal Decline (3.6%), Early High‐Normal Decline (16.5%) and Average (50.5%) (Figure 1). Three trajectories (Early Low‐Rapid Decline, Early Normal‐Rapid Decline and Early Low‐Normal Decline) were associated with increased risk of COPD (‘pre‐COPD’ trajectories).

**FIGURE 1:**
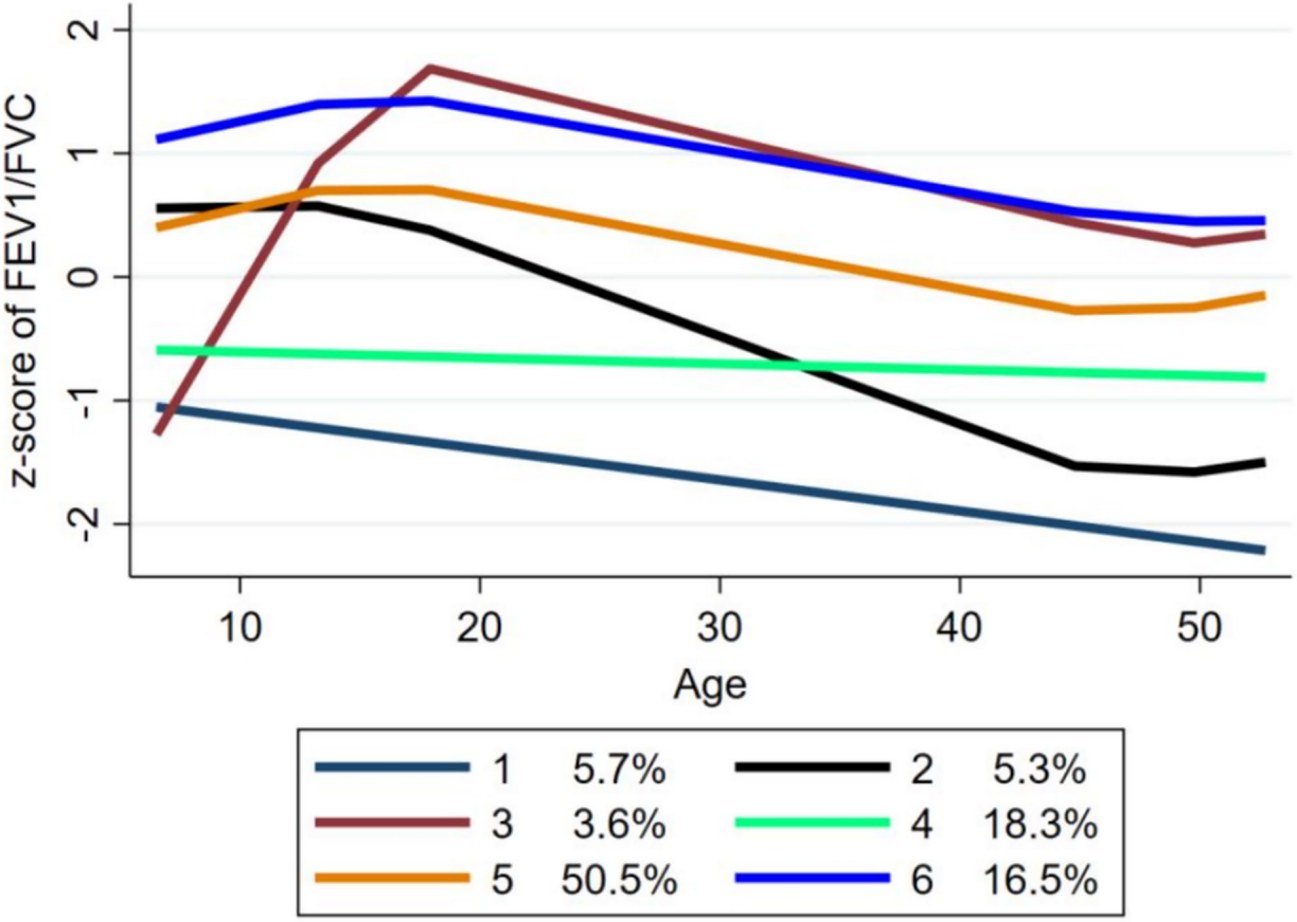
FEV_1_/FVC trajectories Line 1 = ‘Early Low‐Rapid Decline’, line 2 = ‘Early Normal‐Rapid Decline’, line 3 = ‘Early Low‐Catch up‐Normal Decline’, line 4 =‘Early Low‐Normal Decline’, line 5 = ‘Average (normal)’ and line 6 = ‘Early High‐Normal Decline’ trajectories, respectively. Numbers in the box refer to prevalence. Adopted and modified with permit from Ref. [5].

### 2.3 Biomarkers

Blood samples were collected at age 53 years from those study participants from the three pre‐COPD trajectories (Early Low‐Rapid Decline, Early Normal‐Rapid Decline and Early Low‐Normal Decline; *n* = 660) and also from 250 random controls from the Average trajectory. All samples were assayed for a set of 12 selected potential biomarkers based on an extensive literature review of what had been previously measured and found abnormal in clinical airway disease, or which had been implicated in direct immuno‐pathological studies of COPD; that is, having strong biological plausibility. They included: Soluble Receptor for Advanced Glycation End products (sRAGE), fibrinogen, Surfactant Protein D (SPD), Eosinophilic Cationic Protein (ECP), cytokines Interleukins (IL)‐17A, IL‐10, IL‐8, IL‐5, IL‐6, Tumour Necrosis Factor (TNFα), Club‐Cell (CC)‐16, and C‐Reactive Protein (CRP). Biomarker levels were quantified using ELISA.

### 2.4 Socio‐Demographic and Clinical Characteristics

Childhood characteristics were based on parental questionnaires collected in 1968 when participants were 7 years old. Adult characteristics were obtained from questionnaires in the most recent follow‐up when participants were 53 years old. Definitions of childhood and adult characteristics are detailed in the Supporting Information Appendix.

### 2.5 Statistical Methods

Characteristics of participants across FEV_1_/FVC trajectories were compared using Chi‐squared tests. Median levels of biomarkers across trajectories (two rapid decline trajectories, normal decline trajectory and control average trajectory) were compared [11]. Multinomial regression was additionally used to assess associations between biomarker levels and lung function trajectories, adjusting for age, sex, smoking status and asthma. We additionally controlled for the false discovery rate (FDR) using the Benjamini–Hochberg procedure.

We then explored combinations of biomarkers, that is, profiling using Classification and Regression Tree analysis (CART) [12, 13]. CART is a natural framework for finding sub‐groups that have similar biomarker profiles, and identifies thresholds in biomarker levels that define these profiles (details in the Supporting Information). The combinations of biomarker levels that constitute these profiles can be viewed as pathways that are unique to the identified subgroups, and potentially have aetiological relevance. Based on outputs of CART, we also developed score charts demonstrating the link between relevant biomarkers profiles and the distribution of lung function trajectories.

## 3 Results

### 3.1 Characteristics of Study Participants

Among participants included in this analysis, 49.9% were males, 21.3% current smokers, 27.1% obese, and 17.1% had current asthma at age 53 years.

Compared to the Average trajectory, the Early Low‐Rapid Decline trajectory had a higher prevalence of males, current smokers, and respiratory morbidities including asthma, COPD, reduced FEV_1_, respiratory symptoms, medication use, and atopy at 53 years, as well as childhood hay fever and parental smoking (Table 1). Compared to the Average trajectory, the Early Normal‐Rapid Decline trajectory had a higher prevalence of current smoking, higher socioeconomic status (SES), respiratory morbidities including asthma, COPD, reduced FEV_1_, respiratory symptoms, and medication use at 53 years, as well as childhood hay fever, eczema, and parental smoking. The Early Low‐Normal Decline trajectory had a higher prevalence of COPD at 53 years than the Average trajectory. Among the three obstructive pre‐COPD trajectories, the Early Low‐Rapid Decline had the highest prevalence of current smoking, clinical respiratory conditions at 53 years, childhood hay fever, and paternal smoking.

**TABLE 1:** Prevalence (%) of characteristics of study participants across trajectories.

Characteristics	FEV_1_/FVC trajectories			
	Early low‐rapid decline, *n* = 126	Early normal‐rapid decline, *n* = 120	Early low‐normal decline, *n* = 414	Average, *n* = 250 (Reference)
Male sex	60.3**↑	50.8	49.7	44.5
Smoking by 53 years				
Never	26.2	30.0	45.1	48.9
Past	35.7	37.5	37.2	37.5
Current	38.1***↑	32.5***↑	17.7	13.5
SES at 53 years				
1 (lowest)	30.6	28.7*↓	34.3	38.7
2	13.7	9.6	14.9	13.6
3	37.1	36.5	33.3	34.9
4	7.3	11.3	7.3	4.9
5	11.3	13.9	10.1	7.8
Obesity by 53 years	23.0	21.7*↓	27.2	31.8
Clinical characteristics at 53 years				
Ever asthma	53.1***↑	35.0*↑	25.6	24.6
Current asthma	45.2***↑	20.8**↑	11.6	10.2
COPD (LLN)	54.4***↑	21.8***↑	2.4	0.82
COPD (GOLD)	77.6***↑	36.9***↑	4.8*↑	1.6
FEV_1_ < 80% predicted^a^	32.0%*** ↑	21.0%*** ↑	4.8%	2.5%
Usual cough	36.8**↑	27.9	26.1	24.0
Usual sputum	22.7	19.6	13.1	15.9
Shortness of breath at rest	16.8**↑	12.7*↑	9.4	6.1
Shortness of breath after exercise	37.6***↑	29.1**↑	17.9	18.3
Woken due to chest tightness	18.4 ** ↑	14.2	8.5	8.9
Woken due to shortness of breath	16.7 ** ↑	15.3 * ↑	8.0	8.1
Any medicine for breathing in the last year	44.8***↑	23.9***↑	12.5	10
Atopy	72.1%* ↑	56.4%	58.2%	58.7%
Childhood characteristics				
Childhood asthma	36.2	22.5*↓	23.4**↓	33.2
Pneumonia	19.5	12.6	16.4	17.0
Hay fever	24.5**↑	19.3*↑	14.8	11.1
Eczema	20.5	24.3*↑	14.7	15.5
Maternal asthma	17.3	12.1	11.0	10.6
Paternal asthma	16.0	12.7	12.1	11.6
Parental asthma	28.9	20.8	21.6	20.5
Maternal smoking	42.1	46.9**↑	32.2	33.6
Paternal smoking	65.8**↑	68.1**↑	53.9	48.7
Low birth weight^b^	6.1	7.7	6.0	10.6
Preterm^b^	20.7	13.6	13.4	18.4
Profiles of childhood factors (Ref. [14])				
Infrequent asthma, bronchitis	8.0	14.1	11.7	16.7
Frequent asthma, bronchitis	18.5	6.7	11.4	13.4
Parental smoking	18.5	28.3	17.5	13.8
Allergy	8.0	10.8	12.4	8.1
Frequent asthma, bronchitis, allergy	36.2	35.8	44.8	44.0
Unexposed or least exposed	10.4	4.1	1.9	3.6

*Note:* *p < 0.05; **p < 0.01; ***p < 0.001; *p*‐values were from Chi‐squared tests.

^a^ Derived from GLI reference values.

^b^ Data only available for a subsample.

### 3.2 Levels of Biomarkers Across Pre‐COPD Lung Function Trajectories

Compared to the Average trajectory, the Early Low‐Rapid Decline trajectory had significantly lower levels of sRAGE and CC16, but significantly higher levels of SPD (Table 2). The Early Normal‐Rapid Decline trajectory also had significantly lower levels of sRAGE and CC16, but significantly higher levels of SPD and ECP than the Average trajectory. Compared to the Average trajectory, the Early Low‐Normal Decline trajectory had a significantly lower level of sRAGE. Levels of CRP and fibrinogen were not significantly different between trajectories.

**TABLE 2:** Circulating biomarker levels at 53 years across FEV_1_/FVC trajectories.

	Early low‐rapid decline	Early normal‐rapid decline	Early low‐normal decline	Average (Reference)
sRAGE, median (P25, P75), pg/mL	679.8 (552.8, 919)** **↓**	684.9 (577.6, 902.4)** **↓**	688.1 (568.6, 849)*** **↓**	769.1 (627.4, 963.2)
CC16, median (P25, P75), ng/mL	5.2 (3.6, 6.6)** **↓**	5.3 (3.6, 9.0)* **↓**	6.5 (4.4, 9.5)	6.9 (4.9, 9.5)
CRP, median (P25, P75), μg/mL	2.4 (1.1, 5.8)	2.8 (1.4, 6.1)	2.3 (1.0, 5.3)	2.5 (1.2, 5.9)
ECP, median (P25, P75), ng/mL	11.0 (1.5, 28.1)	14.8 (1.5, 28.5)*^a^ **↑**	12.9 (1.5, 26.6)	14.0 (1.5, 27.2)
Fibrinogen, median (P25, P75), mg/mL	6.8 (5.4, 7.9)	6.5 (5.5, 7.4)	6.4 (5.4, 7.5)	6.3 (5.5, 7.7)
SPD, median (P25, P75), ng/mL	117.4 (72.7, 174.4)* **↑**	109.8 (74.6, 143.2)^a^ ↑	94.5 (61.5, 131.5)	96 (65.3, 147)
IL17A, median (IQR), pg/mL	1.2 (0.5, 1.9)	1.2 (0.6, 2.0)* **↑**	0.9 (0.5, 1.6)	0.9 (0.4, 2.3)
IL5, median (P25, P75), pg/mL	57 (31, 85)	60 (34, 87)	47 (29, 70)	50 (24, 94)
IL10, median (P25, P75), pg/mL	0.48 (0.31, 0.67)* **↑**	0.48 (0.33, 0.68)* **↑**	0.40 (0.29, 0.58)	0.40 (0.26, 0.73)
IL8, median (P25, P75), pg/mL	2.2 (1.1, 7.3)	2.6 (1.3, 11.4)	2.6 (1.1, 13.0)	2.7 (1.2, 15.2)
TNFa, median (P25, P75), pg/mL	4.3 (2.3, 7.4)	5.3 (2.8, 7.8)** **↑**	3.6 (1.9, 6.1)	4.0 (1.6, 8.3)

*Note:* *, **, *** different from the average trajectory; *p < 0.05; **p < 0.01; ***p < 0.001. *p*‐values were generated using median regression.

^a^ Not significant after control for false discovery rate.

Regarding cytokines, compared to the Average trajectory, the Early Low‐Rapid Decline trajectory had significantly higher levels of IL‐10 with a similar trend for IL‐17A and IL‐5. Compared to the Average trajectory, the Early Normal‐Rapid Decline trajectory also had significantly higher levels of IL‐17A, IL‐10, and TNFα with a similar trend for IL‐5. The Early Low‐Normal Decline and Average trajectories had similar cytokine levels (Table 2).

Table 3 compares the levels of biomarkers for rapid decline trajectories (two rapid decline trajectories combined) with the Early Low‐Normal decline and the Average trajectories. Compared to the Average trajectory, the two combined rapid decline trajectories had significantly lower levels of sRAGE, CC16, but higher levels of SPD, IL17A, IL5, IL10 and TNFα (Figure 2). Furthermore, the combined rapid decline trajectories also had significantly lower levels of CC16 and higher levels of SPD, IL‐17A, IL‐5, IL‐10 and TNFα than the Early Low‐Normal decline trajectory. Adjusted regression showed consistent findings for associations between biomarker levels and lung function trajectories (Table S1).

**TABLE 3:** Circulating biomarker levels at 53 years across FEV_1_/FVC trajectories (two rapid decline trajectories were combined).

	Rapid decline trajectories combined	Early low‐normal decline trajectory	Average trajectory (Reference)
sRAGE, median (P25, P75), pg/mL	685 (567, 911)*** **↓**	688.1 (568.6, 849)*** **↓**	769.1 (627.4, 963.2)
CC16, median (P25, P75), ng/mL	5.2 (3.6, 8.2)***^a^ **↓**	6.5 (4.4, 9.5)	6.9 (4.9, 9.5)
CRP, median (P25, P75) μg/mL	2.6 (1.3, 6.1)	2.3 (1.0, 5.3)	2.5 (1.2, 5.9)
ECP, median (P25, P75), ng/mL	13.1 (1.5, 28.1)	12.9 (1.5, 26.6)	14.0 (1.5, 27.2)
Fibrinogen, median (P25, P75) mg/mL	6.6 (5.5, 7.7)	6.4 (5.4, 7.5)	6.3 (5.5, 7.7)
SPD, median (P25, P75), ng/mL	114.2 (74.3, 160)**^a^ **↑**	94.5 (61.5, 131.5)	96 (65.3, 147)
IL17A, median (P25, P75), pg/mL	1.2 (0.57, 1.9)**^a^ **↑**	0.9 (0.5, 1.6)	0.9 (0.4, 2.3)
IL5, median (P25, P75), pg/mL	59 (32, 85)*^a^ **↑**	47 (29, 70)	50 (24, 94)
IL10, median (P25, P75), pg/mL	0.48 (0.32, 0.68)**^a^ **↑**	0.40 (0.29, 0.58)	0.40 (0.26, 0.73)
IL8, median (P25, P75), pg/mL	2.5 (1.2, 9.1)	2.6 (1.1, 13.0)	2.7 (1.2, 15.2)
TNFα, median (P25, P75), pg/mL	4.7 (2.5, 7.5)^a^ **↑**	3.6 (1.9, 6.1)	4.0 (1.6, 8.3)

*Note:* *, **, *** different from the Average trajectory; *p < 0.05; **p < 0.01; ***p < 0.001. *p*‐values were generated using median regression.

^a^ Different from the early low‐normal decline trajectory.

**FIGURE 2:**
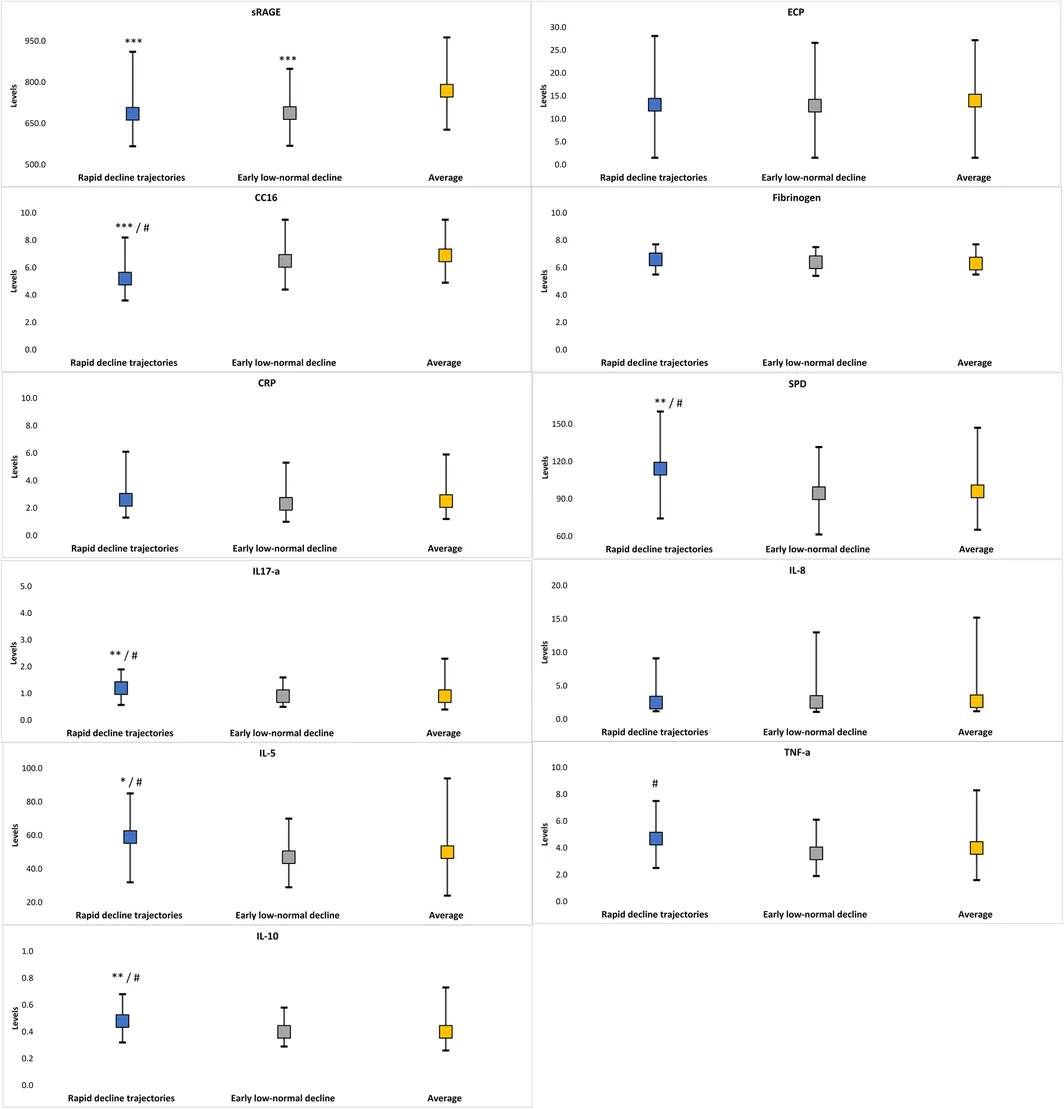
Levels of biomarkers (median [middle box] and Interquartile range [lower and upper bars]) across FEV_1_/FVC trajectories. *, **, *** different from the Average trajectory; *p < 0.05; **p < 0.01; ***p < 0.001; # different from the Early Low‐Normal Decline trajectory. Units for biomarkers: sRAGE (pg/mL), CC16 (ng/mL), CRP (μg/mL), ECP (ng/mL), Fibrinogen (mg/mL), SPD (ng/mL), IL‐17A (pg/mL), IL‐5 (pg/mL), IL‐10 (pg/mL), IL‐8 (pg/mL), TNFα (pg/mL).

After accounting for the false discovery rate, most of the main associations remained statistically significant (see footnotes of Tables 1 and 2).

### 3.3 Potential Biomarker Profiles and Lung Trajectories

Of the biomarkers assessed, the CART indicated that six (CC16, IL‐5, IL‐8, IL‐17A, sRAGE and SPD) may be more important in distinguishing between the pre‐COPD lung function trajectories. Specifically, CART identified a threshold for each biomarker and eight distinct hierarchical combinations/profiles of these biomarkers (Figure S3). The distribution of lung function trajectories varied according to the levels and profiles of these biomarkers (Figure S3, Table S2). These CART‐derived biomarker profiles were summarised into a score chart to facilitate interpretation (Figure S3; details in the Supporting Information).

## 4 Discussion

This study investigated biologically plausible blood biomarkers in middle age that distinguish pre‐COPD lung function trajectories from controls, and rapid decliners from a more stable, early onset pre‐COPD group. CC16, sRAGE, SPD and the cytokines IL‐17A, IL‐5 and IL‐10 significantly differed between pre‐COPD trajectories and the normal/average trajectory. Notably, within the pre‐COPD trajectories, these biomarkers also differed between rapidly progressive decline trajectories and the relatively stable, normal decline one. Our analysis also identified combinations/profiles of these biomarkers at certain levels that could distinguish pre‐COPD trajectories.

To date, studies have focused on identifying biomarkers associated to well‐established COPD. Previous studies have provided some evidence that sRAGE, CC16, SPD, CRP and some cytokines are linked to lower lung function and rapid lung function decline in individuals with COPD, as well as reduced peak lung function in young populations [9, 15, 16, 17, 18, 19, 20, 21]. For example, the ECLIPSE study [16] reported biomarkers but it focused on established COPD patients and lung function progression but did not examine the earlier lung function trajectories that lead to COPD. In contrast, our study is the first to investigate whether established COPD biomarkers are already active during the pre‐COPD phase and can distinguish pre‐COPD lung function trajectories.

The detection of these biomarkers in pre‐COPD trajectories also provides evidence of underlying pathobiological processes in pre‐COPD and suggests their potential role in disease development. Specifically, our findings suggest that some COPD‐specific pathobiological processes including inflammation are already active during the pre‐COPD phase. Notably, the link between biomarkers and lung function trajectories was independent of asthma and smoking. These findings are novel and of significance in the context of growing interest in both detection of pre‐COPD and establishing interventions aimed at slowing or halting the progression of pre‐COPD to full‐blown COPD. For example, early evidence of potential pathobiological processes underpinning pre‐COPD could inform further research into drug targets and the development of preventive therapeutic strategies.

Among analysed biomarkers, CC16 and SPD are both produced and act at the level of the bronchial lumen, whereas IL‐5 and IL‐17A are systemic cytokines that elicit specific immunological responses [22, 23, 24, 25], while others are controllers of inflammatory responses. IL‐5 and IL‐17A have also been implicated in the pathophysiology of asthma—the former as a marker of eosinophilic inflammation and the latter of neutrophilic inflammation [26]. Thus, our CART analysis of this combination of biomarkers could potentially provide unique insight into pathophysiological mechanisms contributing to lung function decline. Our data show that cytokine‐mediated inflammation as indicated by higher levels of markers IL‐5, IL‐17A and IL‐8 is relevant to lung function decline when CC16 is low. Low CC16 is likely due to direct damage to the airway epithelium [27]. Therefore, we hypothesise that certain lung function trajectory phenotypes are related to combinations of both epithelial damage and immune‐driven inflammation.

On the other hand, in individuals with high CC16, possibly suggesting functionally intact airway epithelium, elevated SPD seems to be important in rapid lung function decline, especially when there is concurrent Type 2 inflammation (with high IL‐5). SPD is a newly identified biomarker secreted by the bronchial epithelium, with limited data on its relationship with respiratory disease or lung function decline [23], but is elevated in response to respiratory infections and secondary destructive reactions such as obliterative bronchiolitis [28]. Notably, however, these studies were of lung transplant patients and so their relevance to a more general population, as in our study, is unclear.

In adult patients presenting clinically with symptoms, lung function testing should ideally be a routine investigation. Unfortunately, the vast majority of such patients are unlikely to have had serial measures when well, prior to presentation. Detecting low lung function at only one point in time fails to provide informative context regarding time of commencement of abnormally rapid physiological decline. Our study has identified six biomarkers (IL‐5, IL‐17A, IL‐8, sRAGE, CC16 and SPD) that, in combination, could be potentially valuable for distinguishing lung function trajectory in clinical practice, even in currently well persons as part of health checks (as there are for other common disease entities of later life). This would have two potential benefits: 1. identifying those with abnormally rapid lung function decline, indicating the need for urgent cessation of harmful exposures such as smoking, and 2. identifying potential therapeutic targets that can be trialled.

The main strength of our study stems from the uniqueness of having repeated lung function measures from the first to the sixth decade in TAHS. In addition, the use of an objective data‐driven analytical technique enabled us to identify lung function trajectories as well as explore potential currently unknown relationships between lung function trajectories and biomarker combinations.

However, this study also has some limitations. There were differences in the equipment and technical standards for performing spirometry measurements over time, but this was unlikely to substantially influence the relative positions between trajectories which were based on z‐scores. Healthy‐survivor bias was possible, given that the analysis was limited to those who were alive and continuing to participate in the study, but the differences in childhood lung function and demographic and health characteristics between participants and those deceased or lost to follow up were minimal [5]. If attrition was differential across trajectories, it might influence the lung function trajectory prevalence estimates and generalisability of these estimates, but would be unlikely to affect the actual derived trajectory groups or the associations between these and the biomarkers. There is overlap in biomarker concentrations across trajectories. In practice, this degree of overlap poses challenges for developing clinically meaningful reference limits or diagnostic thresholds. Well‐validated algorithms may be useful in practice.

In this study, we characterised biomarkers for lung function trajectories cross‐sectionally. One limitation of this study is that spirometry was measured at the same time or earlier than biomarkers. Future work will focus on developing formal predictive algorithms that integrate both clinical measures and biomarkers using data from future waves of lung function follow‐up.

As TAHS participants were almost exclusively Caucasian, generalising the findings to other populations should be done with some caution. Finally, the analysis involved multiple biomarkers, raising the possibility of chance findings; we were able to adjust for false discovery rate. However, the findings should be interpreted as hypothesis‐generating, and replication in independent cohorts with comparable life‐course data will be essential. However, the major barrier to such replication studies is that it will take time for them to mature, and they would need to cover both lung function growth and decline phases. Hence, TAHS will likely remain the best available source of evidence for some time.

In conclusion, we show that several biomarkers, including CC16, sRAGE, SPD and several cytokines, present in middle age, differ significantly across pre‐COPD lung function trajectories. These findings suggest that COPD‐specific pathobiological processes, such as lung damage and inflammation, are already active during the pre‐COPD phase. Importantly, our findings underscore the potential of these biomarkers for early detection and risk stratification. This opens the door to establishing validated algorithms based on both biomarkers and clinical history, with the ultimate goal of translating such tools into routine clinical practice for earlier and more targeted prevention of COPD.

## Author Contributions

**Jonathan Pham:** writing – review and editing, software, methodology, investigation. **E. Haydn Walters:** investigation, conceptualization, validation, funding acquisition, data curation, writing – review and editing. **Shyamali C. Dharmage:** conceptualization, funding acquisition, writing – review and editing, methodology, supervision, data curation. **Dinh S. Bui:** conceptualization, investigation, funding acquisition, writing – original draft, formal analysis. **Don Vicendese:** investigation, methodology, formal analysis, writing – review and editing, software. **Nur Sabrina Idrose:** writing – review and editing, methodology, investigation. **Adrian Lowe:** conceptualization, funding acquisition, data curation, methodology, writing – review and editing. **Raisa Cassim:** conceptualization, investigation, funding acquisition, writing – review and editing, data curation. **Garun Hamilton:** investigation, methodology, writing – review and editing, software, data curation. **Caroline Lodge:** writing – review and editing, funding acquisition, conceptualization, methodology, data curation. **Alan James:** investigation, methodology, writing– review and editing, resources, data curation. **Richard Wood‐Baker:** investigation, writing – review and editing, methodology, resources, data curation. **Graeme Zosky:** investigation, methodology, writing – review and editing, data curation. **Paul Thomas:** investigation, methodology, writing – review and editing, resources, data curation. **Michael J. Abramson:** investigation, funding acquisition, writing – review and editing, methodology, supervision. **Peter Frith:** investigation, writing – review and editing, methodology, data curation. **Philip M. Hansbro:** investigation, methodology, writing – review and editing, data curation. **Rosa Faner:** investigation, writing – review and editing, methodology, supervision. **Alvar Agusti:** investigation, methodology, writing – review and editing, supervision. **Jennifer Perret:** investigation, funding acquisition, writing – review and editing, methodology, data curation. **Gary Anderson:** investigation, writing – review and editing, methodology, resources. **Chamara Senaratna:** investigation, writing – review and editing, methodology, software. **Gayan Bowatte:** investigation, writing – review and editing, methodology, data curation. **George Washko:** investigation, writing – review and editing, methodology, supervision.

## Funding

TAHS was supported by the National Health and Medical Research Council (NHMRC) of Australia under NHMRC project grant scheme (299901, 1021275) and the NHMRC‐Horizon Europe Grant Scheme is funded by Australian NHMRC (1101313) as part of ALEC (Ageing Lungs in European Cohorts funded by the EU research and innovation programme under grant agreement No. 633212); The University of Melbourne; Clifford Craig Medical Research Trust of Tasmania; the Victorian, Queensland & Tasmanian Asthma Foundations; The Royal Hobart Hospital Research Foundation; Helen MacPherson Smith Trust; and GlaxoSmithKline. S.C.D., A.L., J.P., C.L., D.B., P.M.H. and E.H.W. are supported by the NHMRC of Australia. This biomarkers project was specifically supported by GSK and the University of Melbourne.

## Disclosure

The authors did not use any artificial intelligence–assisted technologies in the preparation of this manuscript.

## Ethics Statement

The study was approved by Human Ethics Review Committees at all participating institutions (The Universities of Melbourne (040375), Tasmania (0403751, H0012710), New South Wales (08094), The Alfred Hospital (1118/04), and Royal Brisbane and Women's Hospital Health Service District (2006/037)) and written informed consent was obtained from all participants.

## Conflicts of Interest

Don Vicendese is an Editorial Board member for Respirology and was excluded from all editorial decision‐making related to this article. The other authors declare no conflicts of interest.

## Supporting information


**Table S1:** Adjusted associations between circulating biomarker levels at 53 years and individual FEV_1_/FVC trajectories.
**Table S2:** Comparing the association between CART pathways and the risk of being on a Rapid Decline trajectory. The OR is based on comparing pathways to Path number 15 as the reference pathway.
**Figure S1:** Heatmap of the biomarkers. Each row represents a participant. The vertical dendogram indicates correlation between the biomarkers. The horizontal dendogram indicates possible clusters of participants based on their biomarker profiles. The colour indicates biomarkers levels that are log transformed and then Z scaled. White indicates mean values, red lower than the mean and blue higher than the mean. Each biomarker was individually scaled. The biomarkers had highly diverse ranges and so Z scaling was done to limit high magnitude ranges dominating the scale in order to assist visual comparison.
**Figure S2:** The lower triangle shows GAM fits to all univariate associations between the biomarkers. The diagonal display density plots for each biomarker. The upper triangle displays the Spearman correlation for each biomarker pair. The graphs were done on the log scale.
**Figure S3:** Top panel: Differentiation of lung function trajectories by biomarkers in CART. Note the boxes at the bottom of the tree represent the final partitioning of all participants based on their shared biomarker level pathways. The four proportions in the boxes represent the prevalence of each trajectory among the participants in the final partitions. The percentages below the boxes are the percentages of the total sample that are in the final partitions. The biomarkers levels are on the natural log scale. Original units for biomarkers: sRAGE (pg/mL), CC16 (ng/mL), CRP (μg/mL), ECP (ng/mL), Fibrinogen (mg/mL), SPD (ng/mL), IL‐17A (pg/mL), IL‐5 (pg/mL), IL‐10 (pg/mL), IL‐8 (pg/mL), TNFα (pg/mL). CART identified cut‐off points for biomarkers (see Supporting Information).

## Data Availability

The data that support the findings of this study are available on request from the corresponding author. The data are not publicly available due to privacy or ethical restrictions.
