# Supplementary material for: Biomarkers of Pre‐COPD Lung Function Trajectories in Middle Age: A Prospective Study From 1st to 6th Decade
Source: Respirology. 2026 Jul 13;31(10):993–1002. doi: 10.1002/resp.70279 (PMC13634688; doi:10.1002/resp.70279)
Supplement: Supplementary file 1 [file RESP-31-993-s001.docx]

**Appendices**

**Biomarkers of pre-COPD lung function trajectories in middle age: a prospective study from 1st to 6th decade**

**Definitions of childhood and adulthood characteristics**

*Childhood factors were defined using the information provided by parents in the 1968 survey when the children were 7 years old:*

Childhood asthma was defined by the question “Has he/she at any time in his/her life suffered from attacks of asthma or of wheezy breathing?”.

Childhood pneumonia was defined by any doctor’s diagnosis of pneumonia or pleurisy.

Childhood eczema was defined by the question “Does he/she have infantile (baby) eczema?” and “Has he/she ever had eczema in the creases of elbows, wrists or knees?”.

Childhood hay fever was defined by the question “Does he/she get attacks of hay fever?”.

Childhood food allergy was defined by the question “Have you have been told by a doctor that he/she is allergic to any foods?”.

Maternal and paternal smoking during childhood was defined by parent’s response to the question “Do you smoke every day (or six days out of seven)?”.

Maternal and paternal asthma was based on their report of any past history of asthma or attacks of wheezing.

Low birth weight was defined as birth weight<2500g.

Preterm birth was defined as gestational age <37 weeks

*Adult factors were defined using information provided by participants at 53 years of age.*

Current asthma at 53 years was defined by an affirmative response to the question “Have you ever had asthma?” plus any asthma symptom or asthma medication used for breathing problems in the last 12 months.

Other respiratory symptoms were based on questionnaires.

Personal smoking was based on information at 53 years and included three categories: never, past and current smoking. Pack-years of smoking were also calculated using the smoking history provided at this time.

Obesity at 53 years was defined as BMI≥30.

Socio-economic status at 53 years was defined by the occupation of the participant and grouped into five categories according to Australian Standard Classification of Occupations.

Atopy was defined as a positive skin prick test (≥ 3mm diameter wheal) to one or more of twelve allergens (house dust mite, cat, *Hormodendrum, Alternaria, Penicillium, Aspergillus*, rye grass, mixed grasses, egg white, peanut, shellfish, cow’s milk).

Methods: CART

Biomarker levels were log transformed to limit skewness and, due to the selection of 250 random controls that enriched the Average trajectory, CART was weighted by the inverse of the proportion of data represented by each trajectory. For each biomarker, cut off points were determined internally by the CART algorithm. Briefly, all candidate split points between ordered adjacent observations were evaluated. At each candidate threshold, the data were partitioned and the resulting child nodes assessed using the Gini impurity criterion. The selected cut-off corresponds to the threshold that maximised the reduction in weighted node impurity in order to optimise predictive separation within the sample. No investigator defined thresholds were imposed.

The hierarchical structure of a classification tree, based on recursive partitioning, is suited to modelling complex and non-linear associations and lends itself to creating a decision tree that may inform clinical management.

**Classification and Regression Tree analysis and risk score chart**Figure S1,2 displays density plots and Spearman correlation and GAM fits for all biomarker bivariate combinations. There were mainly weak to mild correlations between the biomarkers except for: IL-10 with IL-17A, IL-5 and TNFα; IL-17A with IL-5, IL-6 and TNFα; IL-5 with TNFα; and IL-6 with IL-8.

Of the biomarkers assessed, the CART indicated that six of them (CC16, IL-5, IL-8, IL-17A, sRAGE and SPD) may be more important in differentiating between the pre-COPD lung function trajectories. Specifically, CART identified a threshold for each biomarker and eight distinct hierarchical combinations/profiles of these biomarkers (Figure S3).

The probability of belonging to each trajectory was dependent on levels and profiles of these biomarkers (CC16, IL-5, IL-8, IL-17A, sRAGE and SPD) (Figure S3, Table S2). Levels of biomarkers were classified as high or low based on the identified thresholds. Individuals with higher C16 levels (above the identified threshold) generally had a lower probability of belonging to the rapid decline trajectories. On the other hand, those with lower levels of CC16 plus higher levels of IL-5, IL-8 and/or IL-17A had the highest probability of belonging to rapid decline trajectories.

These CART findings on combinations of biomarkers were translated into a risk score chart (Figure 3). The risk/probability of belonging to each specific impaired trajectory for an individual can be predicted by his/her combined biomarker levels. For example, an individual with low CC16, high IL-5, high IL-17A and high sRAGE has a 79% probability of having a rapid decline lung function trajectory. An individual with high CC16, high SPD and low IL-5 is likely (70% probability) to have an Average lung function trajectory.

**Table S1. Adjusted associations between circulating biomarker levels at 53 years and individual FEV_1_/FVC trajectories**

|  | FEV_1_/FVC trajectories: RRR (95%CI) | | |
| --- | --- | --- | --- |
|  | Early low-rapid decline, N=126 | Early normal-rapid decline, N=120 | Early low-normal decline, N=414 |
| **sRAGE**  Q1  Q2-4 combined | Ref  0.55 (0.36,0.84)** | Ref  0.61 (0.40,0.92)* | Ref  0.57 (0.44,0.73)** |
| **CC16**  Q1  Q2-4 combined | Ref  0.54 (0.29,0.98)* | Ref  0.39 (0.21,0.73)** | Ref  0.66 (0.42,1.03) |
| **CRP**  Q1  Q2-4 combined | Ref  0.65 (0.42,1.02) | Ref  1.3 (0.81,2.2) | Ref  0.81 (0.63,1.05) |
| **SPD**  Q1  Q2-4 combined | Ref  1.4 (0.84,2.4) | Ref  1.9 (1.1,3.4)* | Ref  0.79 (0.55,1.1) |
| **Fibrinogen**  Q1  Q2-4 combined | Ref  1.1 (0.66,1.9) | Ref  1.3 (0.75,2.2) | Ref  1.1 (0.73,1.5) |
| **ECP**  Q1  Q2-4 combined | Ref  0.82 (0.50,1.3) | Ref  1.1 (0.68,1.8) | Ref  1.1 (0.76,1.5) |
| **IL17A**  Q1  Q2-4 combined | Ref  1.3 (0.78,2.1) | Ref  1.6 (0.95,2.7) | Ref  1.2 (0.86,1.8) |
| **IL5**  Q1  Q2-4 combined | Ref  1.7 (1.1,2.9)* | Ref  1.9 (1.1,3.2)* | Ref  1.5 (1.1,2.1)* |
| **IL10**  Q1  Q2-4 combined | Ref  1.2 (0.72,2.1) | Ref  1.8 (1.1,3.1)* | Ref  1.1 (0.78,1.6) |
| **IL8**  Q1  Q2-4 combined | Ref  0.79 (0.47,1.3) | Ref  1.1 (0.66,1.9) | Ref  0.90 (0.63,1.3) |
| **TNFa**  Q1  Q2-4 combined | Ref  1.8 (1.1,3.1)* | Ref  2.3 (1.3,4.0)** | Ref  1.3 (0.95,1.9) |

Q1=the lowest quartile; RRR=Relative Risk Ratio; * p < 0.05; ** p < 0.01; ***p<0.001

Average trajectory is the reference group. Adjusted for age, sex, smoking status and current asthma. Additional adjustment for baseline lung function did not materially change the findings.

**Table S2: Comparing the association between CART pathways and the risk of being on a Rapid Decline trajectory. The OR is based on comparing pathways to Path number 15 as the reference pathway.**

| CART  Path No.  (% *) | Odds Ratio**^†^** (95% CI) P value | Higher risk of  rapid decline |  | Lower risk of  rapid decline | Ratio**^†^** (95% CI) P value | CART  Path No. (% ) |
| --- | --- | --- | --- | --- | --- | --- |
| 4 (18%) | 2.9  (1.9, 4.3)  < 0.0001 | **CC16–IL5–IL17a^‡^** |  |  |  |  |
| 6 (2%) | 6.5  (2.1, 22.0)  < 0.0001 | **CC16–IL5–IL17a–sRAGE** |  | **CC16–IL5–IL17a–sRAGE** | 0.5  (0.2, 1.2)  0.14 | 7 (4%) |
| 9 (13%) | 2.2  (1.3, 3.4)  0.001 | **CC16–IL5–IL8** |  | **CC16–IL5–IL8** | 0.2  (0.01, 1.0)  0.12 | 10 (2%) |
| 13 (14%) | 3.1  (2.0, 4.9)  < 0.0001 | **CC16–SPD–IL5** |  | **CC16–SPD–IL5** | 0.34  (0.02, 1.8)  0.30 | 14 (1%) |
|  |  |  |  | **CC16–SPD** | 1  (ref.) | 15 (46%) |
| ^*^% of participants in the pathway.  **^†^**Odds Ratio compares the within pathway odds of a Rapid Decline trajectory (Early Low or Early Normal) to a non-Rapid Decline (Normal Decline or Average) using pathway 15 as the reference. Within Pathway 15, 60% of participants had a non-Rapid Decline trajectory and represented 46% of all observations.  **^‡^Blue** and **red** indicate low and high levels of a biomarker, respectively, as determined by the CART thresholds in Figure 3. **Blue** for above the threshold and **red** for below. | | | | | | |

**Figure S1**: Heatmap of the biomarkers. Each row represents a participant. The vertical dendogram indicates correlation between the biomarkers. The horizontal dendogram indicates possible clusters of participants based on their biomarker profiles. The colour indicates biomarkers levels that are log transformed and then Z scaled. White indicates mean values, red lower than the mean and blue higher than the mean. Each biomarker was individually scaled. The biomarkers had highly diverse ranges and so Z scaling was done to limit high magnitude ranges dominating the scale in order to assist visual comparison.

**Figure S2**: The lower triangle shows GAM fits to all univariate associations between the biomarkers. The diagonal display density plots for each biomarker. The upper triangle displays the Spearman correlation for each biomarker pair. The graphs were done on the log scale.

There were mainly weak to mild correlations between the biomarkers except for: IL-10 with IL-17A, IL-5 and TNFa; IL-17A with IL-5, IL-6 and TNFa; IL-5 with TNFa; and IL-6 with IL-8.

**Figure S3**. Top panel: Differentiation of lung function trajectories by biomarkers in CART. Note the boxes at the bottom of the tree represent the final partitioning of all participants based on their shared biomarker level pathways. The four proportions in the boxes represent the prevalence of each trajectory among the participants in the final partitions. The percentages below the boxes are the percentages of the total sample that are in the final partitions. The biomarkers levels are on the natural log scale. Original units for biomarkers: sRAGE (pg/mL), CC16 (ng/mL), CRP (μg/mL), ECP (ng/mL), Fibrinogen (mg/mL), SPD (ng/mL), IL-17A (pg/mL), IL-5 (pg/mL), IL-10 (pg/mL), IL-8 (pg/mL), TNFα (pg/mL). CART identified cut-off points for biomarkers (see supplement).

Bottom panel: Risk score chart for the predictive ability of blood biomarkers. EL Rapid=Early Low-rapid decline trajectory; EN Rapid=Early Normal-Rapid Decline trajectory; EL Normal= Early Low-Normal Decline trajectory; Average=Average trajectory. Numbers in the cells represent probability of belonging to a lung function trajectory. For example, an individual with low CC16, high IL-5, high IL-17A and high sRAGE has a 79% probability of having a rapid decline lung function trajectory. Levels of biomarkers (high/low) is based on the threshold identified by CART in the top panel
